# Patellar instability in Indian population: relevance of tibial tuberosity and trochlear groove distance

**DOI:** 10.1051/sicotj/2016008

**Published:** 2016-03-25

**Authors:** Sourabh Kulkarni, Amith P. Shetty, Karan K. Alva, Saurabh Talekar, Vijay D. Shetty

**Affiliations:** 1 Hiranandani Orthopaedic Medical Education (HOME), Dr L. H. Hiranandani Hospital Hillside Avenue Hiranandani Gardens, Powai, Mumbai 400076 India

**Keywords:** TTTG distance, Patellar instability, Patellar dislocation

## Abstract

*Introduction*: The tibial tuberosity to trochlear groove (TTTG) distance in the western population is extensively studied through various modalities such as X-rays, computerised tomography and magnetic resonance imaging. However, to our knowledge there is very little or no literature support to indicate that TTTG distance has been studied in the Indian population.

*Methods*: We therefore undertook a study to measure the TTTG distance in 100 MRI scans of normal Indian knees. Patients with the following co-morbidities were excluded from the study; ligamentous laxity, patellofemoral instability, mal-alignment and osteoarthritis. We measured TTTG distance on the axial MRI slices using OsiriX software.

*Results*: The mean value for females was found to be 14.07 mm and that for male was found to be 13.34 mm. Our study indicates that the TTTG distance, using MRI scans as measurement modality, in the Indian population is significantly different when compared to the published western data.

*Discussion*: We believe that this study can form the basis for future studies on the relationship between TTTG distance and patellar instability in Indian population.

## Introduction

The tibial tuberosity to trochlear groove distance (TTTG distance) is an important parameter to determine the degree of external tibial torsion or lateralisation of the tibial tuberosity. It was initially described by Goutallier et al. in 1978 on axial radiographs taken with the knee in 30 degrees of flexion and neutral rotation [1]. It is known that excessive tibial torsion can result in patellar maltracking and that a TTTG distance greater than 20 mm can lead to patellar instability [2]. TTTG distance in the western population is extensively studied through various modalities such as X-rays, computerised tomography and magnetic resonance imaging (MRI). However to our knowledge, there is very little or no literature support to indicate that TTTG distance has been studied in the Indian population. We therefore undertook a study to measure the TTTG distance in 100 MRI scans of normal skeletally matured Indian knees.

## Material and methods

We reviewed MRI scans of 100 skeletally matured knees in the Indian population. We excluded MRI scans showing any ligamentous injury or any internal derangement of the knee, other than meniscal injury. We also excluded MRI scans of those individuals who, on clinical examination, showed signs and symptoms of patellofemoral instability, ligamentous laxity, malalignment or osteoarthritis as this may give abnormal measurement of the TTTG distance. The MRI scans were done with the individual in the supine position and the knee in full extension. Each measurement of TTTG distance was done by one consultant radiologist and one orthopaedic surgeon.

### Measurement of TTTG distance

All measurements were done by using 1.5 tesla MRI scan. Only axial views were used to measure the TTTG distance in all individuals. We measured the TTTG distance by calculating the horizontal distance between the vertical line passing through the apex of the tibial tuberosity and the vertical line passing through the apex of the trochlear groove as described by Wittstein et al. [3] and Pandit et al. [4]. To avoid inter-observer bias, four readings were taken on each MRI study by two observers. All readings were done using OsiriX software, which runs on Macintosh system (Apple Inc. Cupertino, USA).

**Figure 1.**
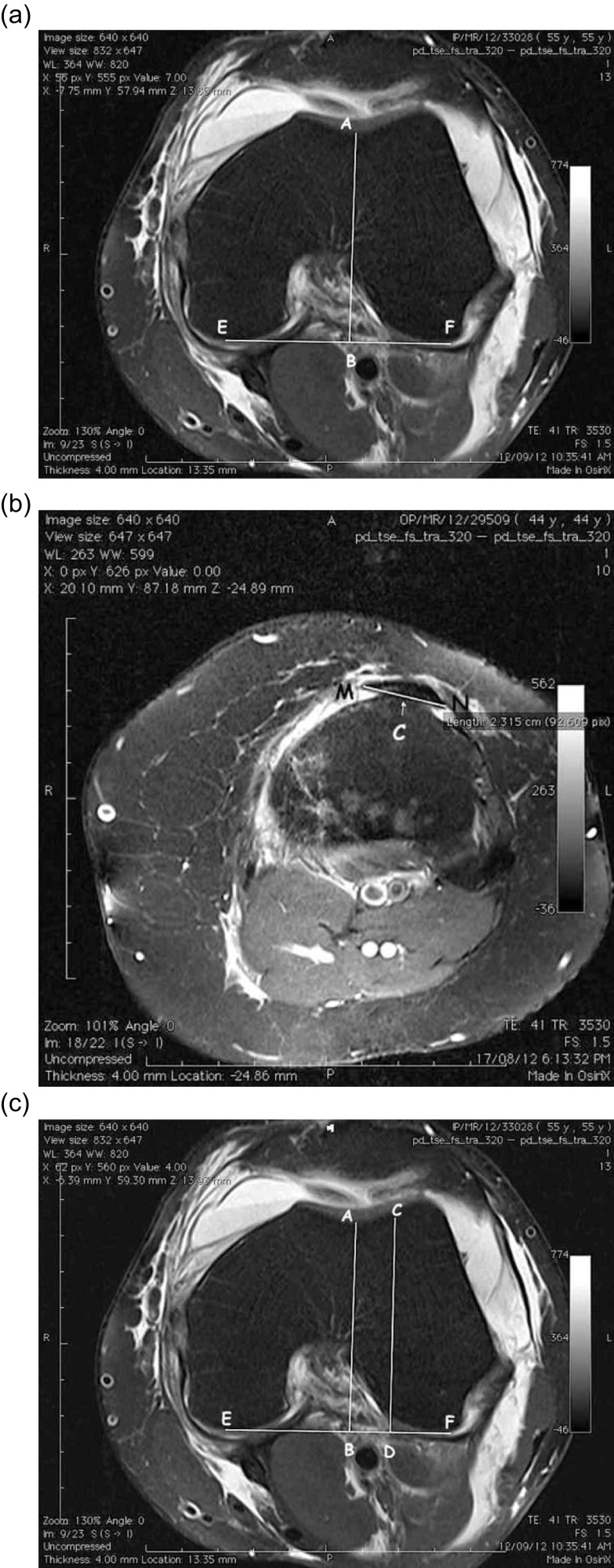
Measurement of TTTG distance using OsiriX software.

To start with the measurement, we first selected an axial image with the deepest trochlear groove (Figure 1a). On this image a posterior condylar line (Line EF) is drawn. A perpendicular line is drawn from the deepest point of the trochlear groove (point A) to line EF in Figure 1a. The images were then scrolled down to the tibial tuberosity until reaching a slice showing the tibial tuberosity with distal most part of the patellar tendon attached to it (Figure 1b). On this slice, the boundaries of the patellar tendon (Line MN, Figure 1b) were marked and the midpoint of this line (point C) represented the tibial tuberosity.

The cursor was kept steady on point C and then images were scrolled up to the previous slice on which markings were done. From the point on which cursor was placed a second perpendicular was drawn on the posterior condylar line (Line CD) as shown in Figure 1c. The distance between these two perpendiculars (distance BD) is calculated as the TTTG distance.

## Statistical analysis

The statistical analysis was carried out using SPSS 16.0 and MS Excel 2007. Statistical analyses tested the null hypotheses of no differences in patients with two groups of different sexes at 95% significance level. A *p* value of less than 0.05 was considered significant (*p* < 0.05). Chi square test and *t* test were also used in the statistical analysis. We applied t test to compare the results of our study with other two studies.

## Results

Table 1 shows demographic data. The coefficient of variation was <10% for both intra and inter-observer analysis. Of the 100 MRI scans, 60 knees were of males and 40 were of females. The mean age of the study group was 37 years (range 20–61 years). The mean TTTG distance was found to be 13.54 ± 6.22 mm (range 7.32–19.76 mm). The mean value for females was found to be 14.07 ± 6.06 mm (range 8.01–20.13 mm) and that for males was found to be 13.34 ± 6.28 mm (range 7.06–19.62 mm; *p* = 0.166, not significant). Our mean values, when compared to published data [3, 4], were found to be significantly different statistically (*p* = 0.001) (Table 2).

**Table 1.** Age and TTTG distance characteristics.

Sex		Age in years	TTTG distance on MAC
F	*N*	40	40
	Minimum	22	6.25
	Maximum	61	19.84
	Range	39	13.59
	Mean	39.20	14.07
	Median	37.00	14.01
	Std. deviation	9.45	3.03
	Std. error of mean	1.49	0.48
M	*N*	60	60
	Minimum	20	6.15
	Maximum	60	21.78
	Range	40	15.63
	Mean	34.77	13.19
	Median	32.00	13.34
	Std. deviation	11.27	3.14
	Std. error of mean	1.46	0.41
Total	*N*	100	100
	Minimum	20	6.15
	Maximum	61	21.78
	Range	41	15.63
	Mean	36.54	13.54
	Median	34.50	13.54
	Std. deviation	10.75	3.11
	Std. error of mean	1.08	0.31

For TTTG: – *t* test = −1.394, *p* = 0.166, not significant.

**Table 2.** Comparison of all MRI studies.

	Study 1	Study 2	Study 3
Author	Pandit et al.	Wittstein et al.	Our study
Year	2011	2006	2012
Sample size	100	20	100
Mean TTTG distance	10	9.4	13.5
*SD*	0.5	0.3	3.1
*t* value*	11.15	12.93	–
*p* value*	< 0.001	< 0.001	–
Significance*	Highly significant	Highly significant	–

* In comparison with our study.

## Discussion

The position of the tibial tubercle is crucial for normal functioning of the quadriceps mechanism because it decides the direction of the inferolateral force vector on the patella and pull of the quadriceps mechanism. Normally, the tibial tuberosity is placed more in line under the femoral sulcus, hence the inferior force vector is much more in magnitude than the lateral force vector. This prevents the lateral subluxation of the patella. But as the tibial tuberosity is placed more laterally, the lateral force vector increases in magnitude, which tries to subluxate or dislocate the patella laterally.

The TTTG distance is one of the major parameters to determine the lateralisation of tibial tuberosity and degree of external tibial torsion. TTTG distance plays a major role in the assessment of patellar instability [5]. It is also used as an indication for a distal realignment procedure for patellar instability [6]. Another factor affecting patellar instability is patella alta. The patellar height can be measured by various methods, the most common being Caton-Deschamps index [7]. Here the distance between the lower border of the articular surface of the patella and the antero-superior border of the tibia is compared with the length of the patellar articular cartilage.

The TTTG distance can be measured accurately with both computerised tomography as well as MRI; however, an MRI scan study has several advantages over CT scan study as it is free of radiation hazards and the MRI scan can evaluate the cartilage damage as a result of recurrent patellar dislocations [8]. In patients with severe trochlear dysplasia requiring trochleoplasty, assessment of the cartilage is pivotal as pre-operative cartilaginous degeneration has been associated with inferior results [5, 9, 10]. MRI has an added advantage of helping in determining the exact centre of patellar tendon attached to tibial tuberosity [11, 12].

There is a wide range of normal TTTG values given in different studies. Using the MRI scan as an imaging modality Wittstein et al. [3] found it as 9.4 ± 0.6 mm and Pandit et al. [4] found it as 9.91 mm in males and 10.04 mm in females. Table 3 shows these studies with their results.

**Table 3.** Studies showing TTTG distance measurement.

Author	Year	Modality	Sample size	Mean TTTG distance	TTTG distance for males	TTTG distance for females
Pandit, Frampton, Stoddart and Lynskey	2011	MRI	100	10 ± 1 mm	9.91 mm (95% CI 8.9–10.8 mm)	10.04 mm (95% CI 8.9–11.1)
Wittstein, Bartlett, Easterbrook and Byrd	2006	MRI	20	9.4 ± 0.6 mm	–	–

In our study, the mean normal value of the TTTG distance in an adult Indian population was found to be 13.54 ± 6.22 mm (males 13.19 ± 6.28 mm and females 14.07 ± 6.06). The difference between the values for males and females was not found to be statistically significant (*p* = 0.166).

As stated in Table 3, we found two studies that used MRI scan as an imaging modality. After comparing our study results with their results we found that the difference in the TTTG distances is statistically significant in our study. Our values indicate that the TTTG distance is far above the values published in the literature. However, it is not clear whether the incidence of patellar dislocation, in the Indian population, is different from that of western population. In our study we used double blindedness of the measurements between two investigators. We also had a uniform method of measurement.

## Conclusion

Our study indicates that TTTG distance, using MRI scans as a measurement modality, in the Indian population is significantly different when compared to the published western data. We do not yet know whether this has an impact on the incidence of patellar instability in the Indian population. We believe that this study forms the basis for further research to study the association between TTTG distance and patellar instability in Indian knees.

## Conflict of interest

The authors declare no conflict of interest in relation with this paper.
